# Ethanol infusion into the vein of Marshall reduced atrial tachyarrhythmia recurrence during catheter ablation: A systematic review and meta-analysis

**DOI:** 10.1016/j.hroo.2024.08.011

**Published:** 2024-09-27

**Authors:** Raymond Pranata, William Kamarullah, Giky Karwiky, Chaerul Achmad, Mohammad Iqbal

**Affiliations:** Department of Cardiology and Vascular Medicine, Faculty of Medicine, Universitas Padjadjaran, Hasan Sadikin General Hospital, Bandung, Indonesia

**Keywords:** Ethanol infusion, Vein of Marshall, Atrial fibrillation, Catheter ablation, Mitral isthmus, Coronary sinus, Marshall bundle, Tachycardia, Alcohol

## Abstract

**Background:**

Ethanol infusion into the vein of Marshall (EIVoM) may increase mitral isthmus bidirectional block (MIBB) and cause local autonomic denervation that may improve outcome.

**Objective:**

This meta-analysis aimed to investigate whether the addition of EIVoM to atrial fibrillation (AF) ablation led to a better outcome.

**Methods:**

Systematic literature search was performed using PubMed, Scopus, ScienceDirect, and Europe PMC for studies that compared the addition of EIVoM during AF ablation with radiofrequency ablation. The primary outcome was *atrial tachyarrhythmia* (ATa) recurrence, defined as AF/atrial flutter/atrial tachycardia after the blanking period.

**Results:**

There were 2821 patients from 11 studies, and EIVoM was successful in 77% (95% confidence interval [CI] 62%–92%). ATa recurrence was 27% (95% CI 20%–34%) in the EIVoM group and 42% (95% CI 33%–51%) in ablation-only group. EIVoM reduced ATa recurrence (odds ratio [OR] 0.52; 95% CI 0.36–0.76; *P* < .001; I^2^ = 76.92). The rate of MIBB was 85% (95% CI 77%–94%) in the EIVoM group and 73% (95% CI 61%–85%) in the ablation-only group, which was significantly higher (OR 3.87; 95% CI 1.46–10.28; *P* < .001; I^2^ = 83.68). The mitral isthmus reconnection rate (OR 0.44; 95% CI 0.15–1.29; *P* = .14; I^2^ = 63.6) and repeat procedure rate (OR 0.76; 95% CI 0.53–1.08; *P* = .12; I^2^ = 48) were similar; however, a leave-one-out sensitivity analysis showed *P* < .05 for both. The benefits of EIVoM were not affected by age, left atrial diameter, and left ventricular ejection fraction (*P* > .05). Age (*P* = .029) and left atrial diameter (*P* = .042) were inversely associated with EIVoM benefits in terms of repeat ablation and mitral isthmus reconnection (age; *P* = .003).

**Conclusion:**

The addition of EIVoM to ablation increased MIBB and reduced ATa recurrence.

**Key Findings**

▪ Ethanol infusion into the vein of Marshall (EIVoM) in addition to catheter ablation increased mitral isthmus bidirectional block during the procedure and reduced atrial tachyarrhythmia recurrence.
▪ Age and left atrial (LA) diameter were inversely associated with EIVoM benefits in terms of repeat ablation and mitral isthmus reconnection.
▪ Whether EIVoM or ablation should be performed first was controversial; on the basis of available data, we can consider performing EIVoM first apart from patients with large LA diameter or advanced age or in a limited resource setting, although more research is needed to address this issue.
▪ The data on ethanol dose were inadequate to confidently draw a conclusion; however, an ethanol injection of ≥5 mL might be required, especially if LA posterior wall isolation is to be performed.

## Introduction

With the advancement of the state-of-the-art medical management of atrial fibrillation (AF), a variety of procedures have emerged, including point-by-point radiofrequency (RF) catheter ablation and cryogenic balloon catheters capable of inducing pulmonary vein isolation (PVI) with a single shot. Currently, constant novel techniques and approaches are still being explored to improve PVI.^1^, ^2^, ^3^, ^4^ However, the recurrence of atrial arrhythmias after PVI remained high, especially for persistent AF.^5^ Contemporary ablation techniques for AF appear to have achieved their “ceiling effect” despite several decades of advancement.^3^ As a result, continual technical breakthroughs are being pursued in fervor to generate sustainable lesions in bidirectional blocks.

The Marshall bundle (MB) is believed to be the primary source of nonpulmonary veins that may be involved in AF. The MB comprises blood vessels (the vein of Marshall), muscle tissue, fat, connective tissue, and nerve tissue, all of which may act as a reentry trigger for atrial arrhythmias, particularly AF, because of their anatomical complexity. In addition to the source of AF triggers, the tract for parasympathetic and sympathetic innervation contributes to AF maintenance.^5^, ^6^, ^7^, ^8^, ^9^ Because of their isolated position within adipose tissue, ablation procedures are unable to completely eradicate the MB structures and adjacent parasympathetic ganglia.^10^^,^^11^ To overcome this limitation, the MB structure can be abolished by ethanol infusion into the vein of Marshall (EIVoM). EIVoM creates chemical trauma that directly damages local neurons, enabling elimination of AF triggers, generating conduction block, and thus mitigating AF triggers.^5^^,^^12^, ^13^, ^14^, ^15^ The aim of this meta-analysis was to summarize the most recent evidence regarding EIVoM in patients with AF. The authors aimed to yield a more elaborate comparison analysis, meta-regression analysis, and a detailed discussion to provide novel and credible insights into this issue.

## Methods

### Protocol and registration

This systematic review was conducted in accordance with the *Cochrane Handbook for Systematic Reviews of Interventions* and reported on the basis of the Preferred Reporting Items for Systematic Reviews and Meta-Analysis (PRISMA). The protocol was registered at the International Prospective Register of Systematic Reviews (PROSPERO), under identification number (CRD42024550495).

### Literature search strategy

**Figure 1:**
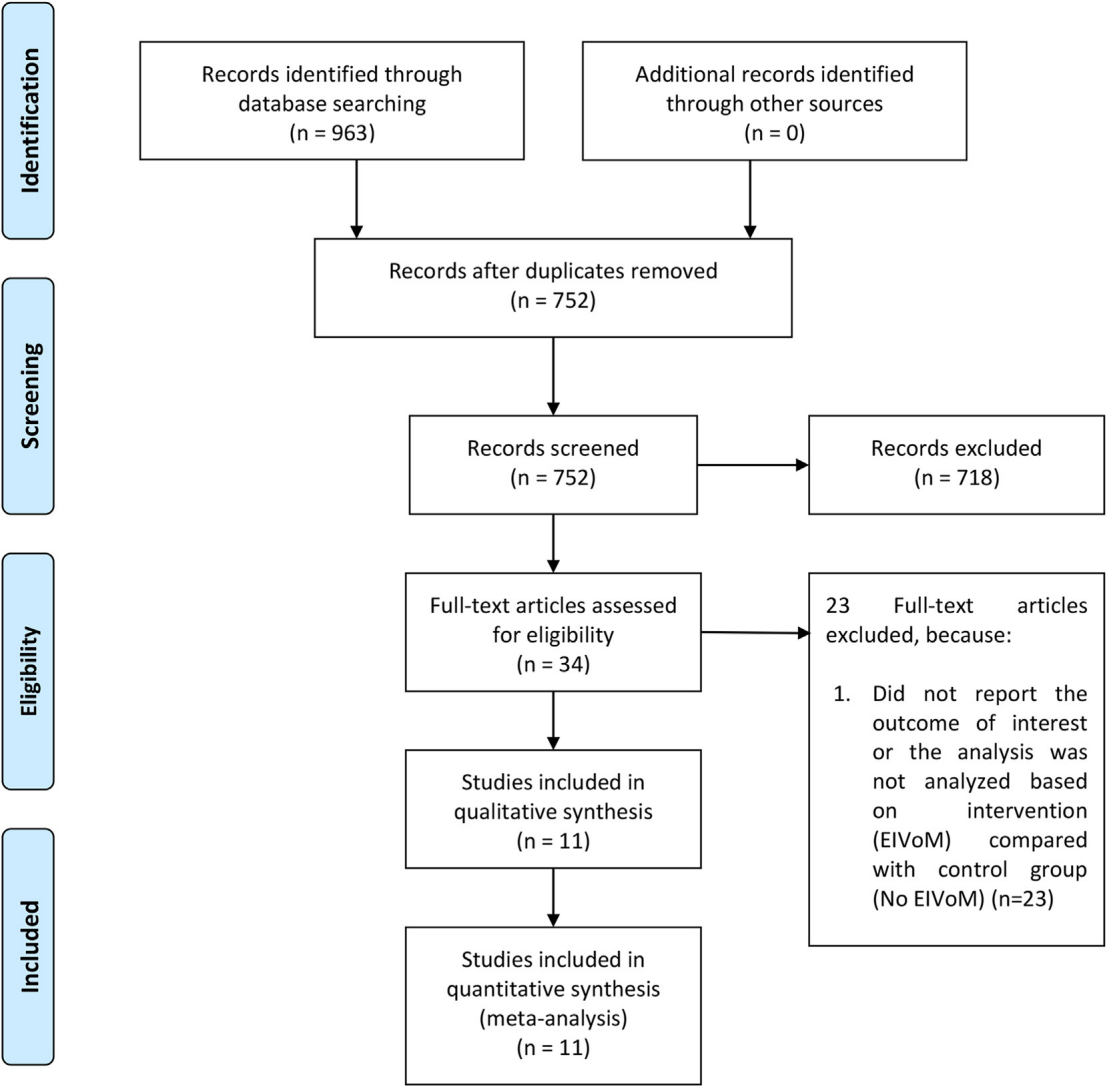
PRISMA flowchart. EIVoM = ethanol infusion into the vein of Marshall.

We examined the databases PubMed, Scopus, Europe PMC, and ScienceDirect up to May 1, 2024. The search terms were as follows: ((ethanol) or (alcohol)) and ((vein of marshall) or (marshall vein) or (ligament of marshall) or (marshall ligament)) and (atrial fibrillation). When required, the reference lists of the included research and relevant review papers were scrutinized for additional references. We tailored the search keywords to the requirements of each database. Our search followed the PRISMA principles, and the flowchart in Figure 1 depicts the search and screening procedures.

### Study selection

We included randomized controlled trials, observational studies (both prospective and retrospective) reporting detailed periprocedural characteristics and outcomes in EIVoM, as well as studies comparing its efficacy and outcomes (atrial tachyarrhythmia [ATa] recurrence, mitral isthmus bidirectional block [MIBB] rate at the most recent available follow-up, rate of reconnection, and repeat ablation procedure) with catheter ablation only in this meta-analysis. Our analysis omitted studies that failed to provide sufficient data. Animal studies, review papers, editorials, comments, letters to editors, case reports/series, meta-analyses, and conference abstracts were also excluded from our meta-analysis.

### Outcomes of interests

The primary outcome of this study was *ATa recurrence*, defined as AF/atrial flutter/atrial tachycardia events lasting >30 seconds after ablation for at least 3 months (blanking period). The secondary outcomes were the MIBB rate, rate of mitral isthmus reconnection, and repeat ablation procedure.

### Data extraction and risk of bias assessment

The course of data abstraction was independently conducted by 2 authors using a form detailing baseline characteristics of the included studies (age, study design, sample size, inclusion, and criteria), type of AF, ethanol dose, left atrial (LA) size, left ventricular ejection fraction (LVEF), follow-up length, and characteristics in repeat ablation.

The Newcastle-Ottawa Scale was implemented by the authors to independently assess the possibility of bias in each observational studies. A study with a total score of ≥7 was deemed bias free. Research with a total score of ≤6 was biased. The Cochrane risk of bias assessment tool was used to assess the risk of bias for randomized controlled trials. Author discussion was used to settle quality rating disagreements.

### Data analysis

In this meta-analysis, we implemented STATA 17 (Stata Statistical Software: Release 18; StataCorp LLC, College Station, TX) to calculate the magnitude of the overall effect. The Mantel-Haenszel method and the generic inverse variance approach were used for dichotomous and continuous data, respectively. Odds ratios (ORs) were used to measure binary comparison. I^2^ was used to measure the pooled estimate’s heterogeneity; a value of >50% or a *P* value of <.10 denotes statistically significant heterogeneity. The random effects model using Sidik-Jonkman method was used for the analyses, regardless of heterogeneity, to calculate the pooled effect size. An REML approach meta-regression analysis was also used to identify any confounders on the basis of the baseline and clinical characteristics of the individuals throughout the incidence and comparison of arrhythmia recurrences between the 2 groups. Sensitivity analyses were performed to test statistical robustness of pooled results, to see whether there is a significant change in pooled results by exclusion of studies, and to single out studies with high heterogeneity. Subgroup analyses were performed for the prospective cohorts and randomized controlled trial subgroup. Furthermore, the Egger test was used to quantify the publication bias. All statistical analyses were 2-sided, with statistical significance attained by a *P* value of <.05.

## Results

There were 2821 patients from 11 studies (2 randomized controlled trials and 9 observational studies) (Figure 1).^5^^,^^16^, ^17^, ^18^, ^19^, ^20^, ^21^, ^22^, ^23^, ^24^, ^25^ EIVoM was successfully performed in 77% (95% confidence interval [CI] 62%–92%) of patients. Failure was mostly due to an absent vein of Marshall or difficulty during cannulation. The baseline characteristics of the studies are outlined in Table 1. The included studies have a Newcastle-Ottawa Scale score of ≥7 for observational studies and low risk of bias for randomized controlled trials. The rate of complications between the EIVoM and RF groups was comparable (Table 2). The rate of VoM-related complications, such as dissection, ranged from 1.5% to 4%.

**Table 1:** Baseline characteristics of the included studies

Study	Design	Sample size	Inclusion criteria	Ethanol volume	Type of AF	EIVoM or ablation first	Age (y)	LA diameter (mm)	LVEF (%)	Mean FU (mo)	FU modality	Characteristics in repeat ablation	NOS score
Gao et al^24^	PO	76 vs 89	Perimitral AT in patients with previous ablation at the MI region	7.4	Perimitral AT	Ablation first (63%) EIVoM first (37%)	62 vs 63	42 vs 43	61 vs 59	12	24-h Holter at 1, 2, 3, and 6 mo	NA	7
Ishimura et al^25^	RO	176 vs 384	Initial MI ablation, combined de novo and repeat ablation	3.8	Nonparoxysmal AF	EIVoM first	67 vs 67	48 vs 48	61 vs 60	12	ECG at 1, 4, 7, 10, and 13 mo and 7-d event recording at 3, 6, and 12 mo	MI reconnection: EIVoM: 25/43 Control: 39/80	7
Ishimura et al^16^	RO	177 vs 236	LAPW and MI ablation	Mean 5.1 ≥5 (60%) <5 (40%)	84% Nonparoxysmal	EIVoM first	69 vs 69	49 vs 49	60 vs 61	13	ECG at 1, 4, 7, 10, and 13 mo and 7-d event recording at 3, 6, and 12 mo	MI-dependent flutter: EIVoM: 3/35 Control: 3/38	8
Lai et al^17^	PO	66 vs 125	De Novo ablation: PVI + roofline, CTI, MI	6.9	Persistent AF	EIVoM first	61 vs 61	44 vs 43	59 vs 59	12	24-h Holter at 1, 2, 3, 6, and 12 mo	Perimitral flutter: EIVoM: 2/4 Control: 3/12	8
Liu et al^18^	PSM RO	32 vs 96	VoM triggers or failed first-attempt endocardial ablation of mitral flutter	2–4	Nonparoxysmal AF	Ablation first	56 vs 56	42 vs 42	58 vs 58	47	ECG, 24-h Holter, or 7-d event recording every 3 mo	NA	8
Nakashima et al^19^	RO	152 vs 110	De novo (in the ethanol group) PVI and posterior MI ablation	NA	Mainly persistent (98%)	EIVoM first	64 vs 61	NA	60 vs 60	9	ECG and 24-h Holter at 1, 3, 6, and 12 mo	MI reconnection: EIVoM: 13/35 Control: 31/46	8
Okishige et al^20^	PO	90 vs 80		4.4	Paroxysmal AF	EIVoM first	63 vs 64	41 vs 39	59 vs 67	12	ECG and 24-h Holter at 1, 2, 3, 6, 9, 12, and 15 mo	No data on MI reconnection Reconnection of >1 PV: EIVoM: 1/17 Control: 21/25	7
Shimizu et al^21^	RO	50 vs 174	PVI and linear ablation including an MI, LA roof, and CTI ablation	4.5	Persistent AF	Ablation first	67 vs 68	46 vs 45	54 vs 54	33	ECG and 24-h Holter at 1, 3, 6, 9, and 12 mo	MI reconnection: EIVoM: 2/7 Control: 18/41	8
Takigawa et al^22^	PO	32 vs 71	Post-AF AT demonstrated perimitral flutter	2–10	Perimitral AT	EIVoM first	63 vs 63	NA	54 vs 56	12	24-h Holter at 1, 3, 6, 9, and 12 mo	Perimitral flutter: EIVoM: 2/5 Control: 8/14 MI reconnection: 2/5 EIVoM: 13/14	8
VENUS trial^5^	RCT	185 vs 158	De novo persistent AF; PVI and isolation of the posterior wall, MI ablation, and ablation of complex fractionated atrial electrogram	5	Persistent AF	EIVoM first	67 vs 66	45 vs 47	52 vs 53	12	ECG at 1, 3, 6, 9, and 12 mo and 1-mo monitoring at 6 and 12 mo	NA	Low risk of bias∗
Zuo et al^23^	RCT	45 vs 44	De novo persistent AF; PVI and roofline ablation + posterior MI ablation + CTI ablation	7 (mean)	Persistent AF	EIVoM first	63 vs 63	42 vs 43	57 vs 58	12	24-h Holter at 3, 6, and 12 mo	MI reconnection: EIVoM: 1/4 Control: 4/8	Low risk of bias∗

AF = atrial fibrillation; AT = atrial tachycardia; CTI = cavotricuspid isthmus; ECG = electrocardiography; EIVoM = ethanol infusion into the vein of Marshall; FU = follow-up; LA = left atrial/atrium; LAPW = left atrial posterior wall; LVEF = left ventricular ejection fraction; MI = mitral isthmus; NA = not available; NOS = Newcastle-Ottawa Scale; PO = prospective observational; PSM = propensity score matched; PV = pulmonary vein; PVI = pulmonary vein isolation; RCT = randomized controlled trial; RO = retrospective observational; VoM = vein of Marshall.

∗ Based on the Cochrane risk of bias assessment tool.

**Table 2:** Complication rates

Study	Sample size	Complications
Gao et al^24^	76 vs 89	EIVoM group: CS or ostium of VoM dissection in 3 patients (3.9%) (uneventful) No cardiac tamponade Asymptomatic minor pericardial effusion in 2 patients (2.6%) RF group: Unknown
Ishimura et al^25^	176 vs 384	EIVoM group: None reported RF group: Pericardial effusion in 4 patients (1%) (2 tamponade)
Ishimura et al^16^	177 vs 236	EIVoM group: CS dissection in 3 patients (1.7%) Pericardial effusion in 1 patient (0.6%) RF group: Pericardial effusion in 1 patient (0.4%)
Lai et al^17^	66 vs 125	EIVoM group: Ostium of VoM dissection in 1 patient (1.5%) Mild pericardial effusion in 1 patient (1.5%) Fluid overload in 1 patient (1.5%) RF group: Mild pericardial effusion in 1 patient (0.8%) Fluid overload in 4 patients (3.2%) Atriovenous fistula in 2 patients (1.6%) Pleural effusion in 1 patient (0.8%)
Liu et al^18^	32 vs 96	EIVoM group: No periprocedural complications RF group: Minor complications in 2 patients (2.1%)
Nakashima et al^19^	152 vs 110	EIVoM group: CS or VoM dissection in 2 patients (1.3%) RF group: Cardiac tamponade in 1 patient (0.91%)
Okishige et al^20^	90 vs 80	EIVoM group: CS dissection in 2 patients (2.2%) Pericardial tamponade in 1 patient (1.3%) RF group: Pericardial tamponade in 3 patients (3.8%)
Shimizu et al^21^	50 vs 174	EIVoM group: VoM dissection in 2 patients (4%) RF group: Pericardial tamponade in 2 patients (1.1%)
Takigawa et al^22^	32 vs 71	EIVoM group: Pericardial effusion in 1 patient (3.1%) RF group: Pericardial effusion in 5 patients (7%)
VENUS trial^5^	185 vs 158	EIVoM group: Pericardial effusion requiring drainage in 2 patients (1.1%) Pericardial effusion not requiring drainage in 11 patients (6.0%) Stroke in 1 patient (0.5%) Transient ischemic attack in 2 patients (1.1%) Fluid overload in 10 patients (5.4%) RF group: Pericardial effusion requiring drainage in 2 patients (1.3%) Pericardial effusion not requiring drainage in 6 patients (3.8%) Stroke in 2 patients (1.3%) Transient ischemic attack in 2 patients (1.3%) Fluid overload in 2 patients (1.3%)
Zuo et al ^23^	45 vs 44	EIVoM group: No VoM rupture or ethanol spillage Pericardial effusion in 2 patients (4.4%) RF group: Pericardial effusion in 1 patient (2.3%)

CS = coronary sinus; EIVoM = ethanol infusion into the vein of Marshall; RF = radiofrequency; VoM = vein of Marshall.

### ATa recurrence

**Figure 2:**
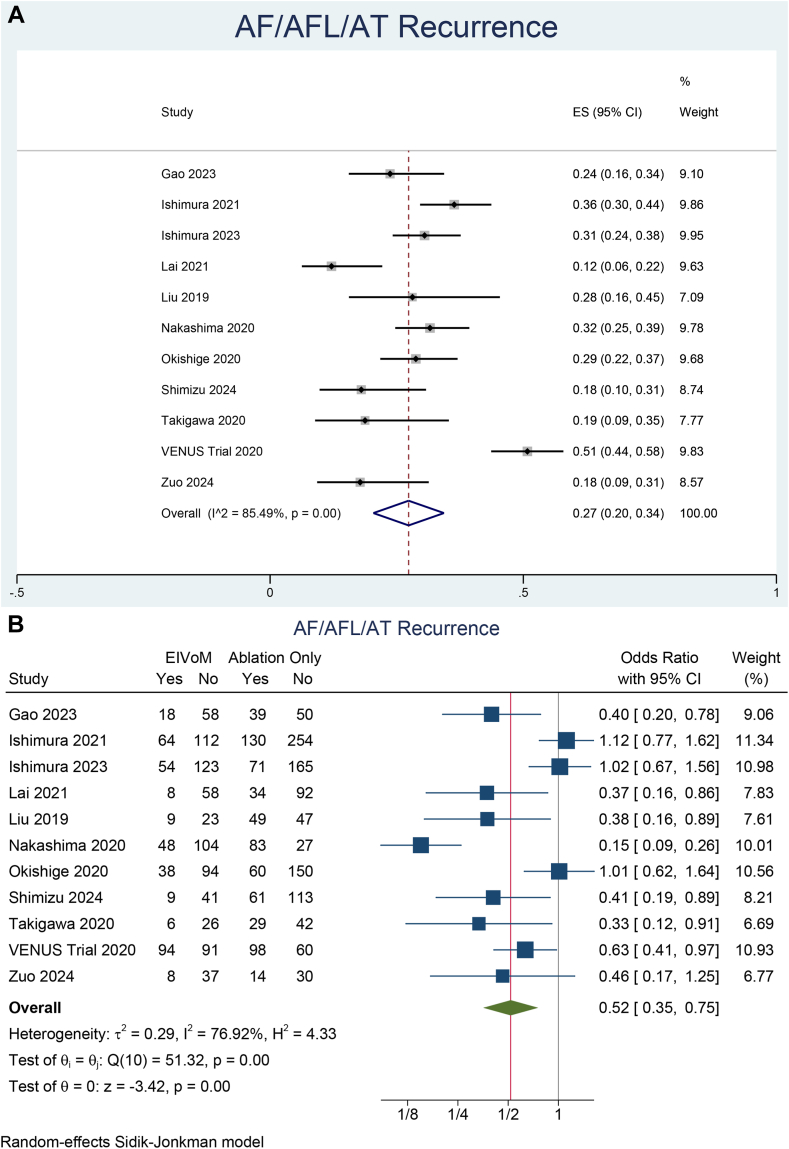
Atrial tachyarrhythmia recurrence. (**A**) Incidence and (**B**) comparison of arrhythmia recurrences between the EIVoM and ablation-only groups. AF = atrial fibrillation; AFL = atrial flutter; AT = atrial tachycardia; CI = confidence interval; EIVoM = ethanol infusion into the vein of Marshall; ES = effect size.

ATa recurrence was 27% (95% CI 20%–34%) in the EIVoM group (Figure 2A). The recurrence was not affected by age (*P* = .154), LA diameter (*P* = .423), and LVEF (*P* = .612). ATa recurrence was 42% (95% CI 33%–51%) in the ablation-only group. EIVoM reduced ATa recurrence compared with RF ablation-only (OR 0.52; 95% CI 0.36–0.76; *P* < .001; I^2^ = 76.92, *P* < .001) (Figure 2B). A leave-one-out sensitivity analysis showed that the reduction remained statistically significant (*P* < .05). A meta-regression analysis showed that the benefits of EIVoM were not influenced by age (*P* = .150), LA diameter (*P* = .157), and LVEF (*P* = .745).

### MIBB

**Figure 3:**
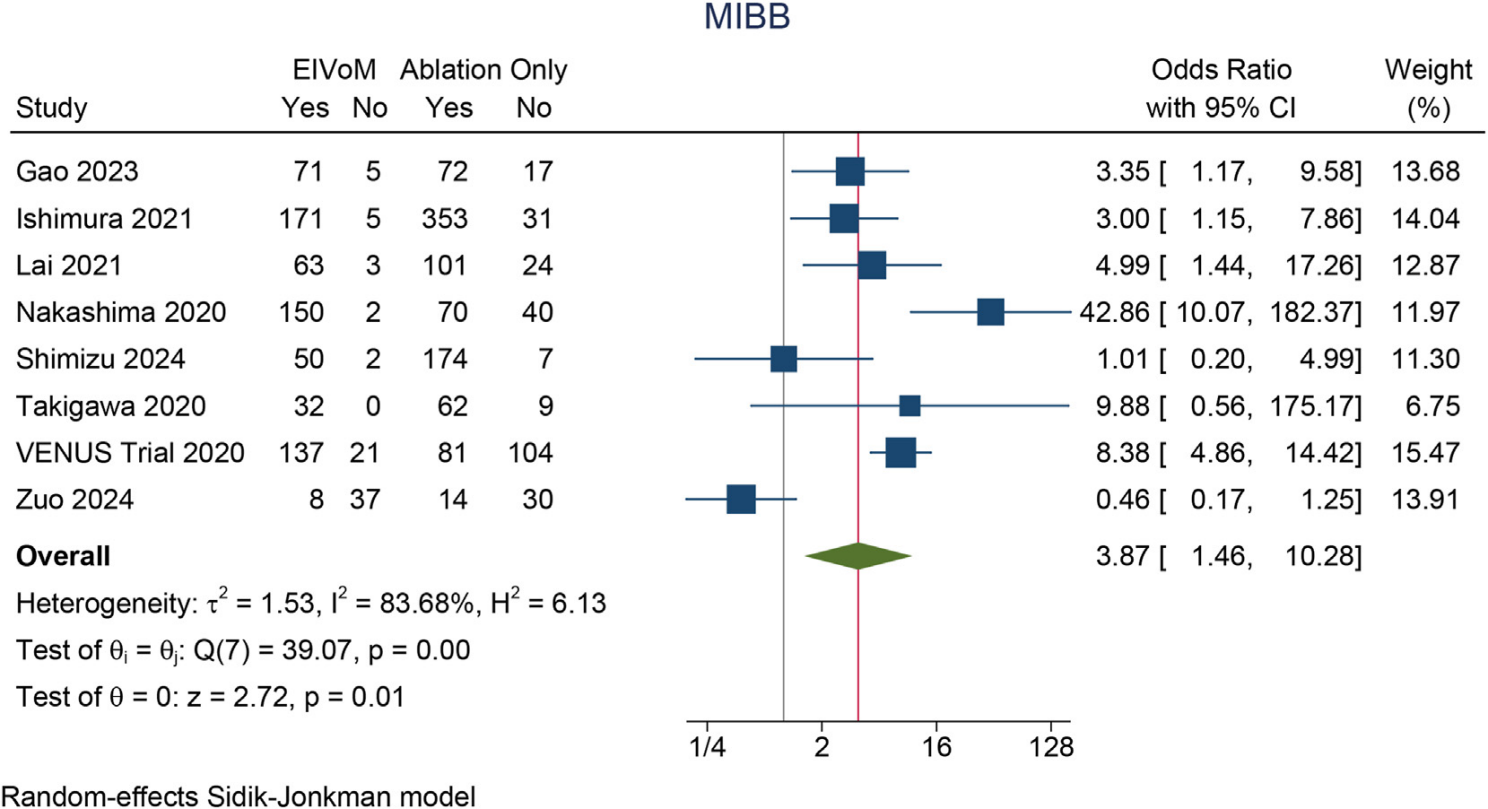
MIBB between the EIVoM and ablation-only groups. CI = confidence interval; EIVoM = ethanol infusion into the vein of Marshall; MIBB = mitral isthmus bidirectional block.

The rate of MIBB was 85% (95% CI 77%–94%) in the EIVoM group and 73% (95% CI 61%–85%) in the ablation-only group. The rate of MIBB in the EIVoM group was significantly higher than that in the ablation-only group (OR 3.87; 95% CI 1.46–10.28; *P* < .001; I^2^ = 83.68, *P* < .001) (Figure 3). A leave-one-out sensitivity analysis showed that the higher rate of MIBB remained statistically significant (*P* < .05). A meta-regression analysis showed that the benefits of EIVoM were not influenced by age (*P* = .962), LA diameter (*P* = .564), and LVEF (*P* = .826).

### Repeat ablation

**Figure 4:**
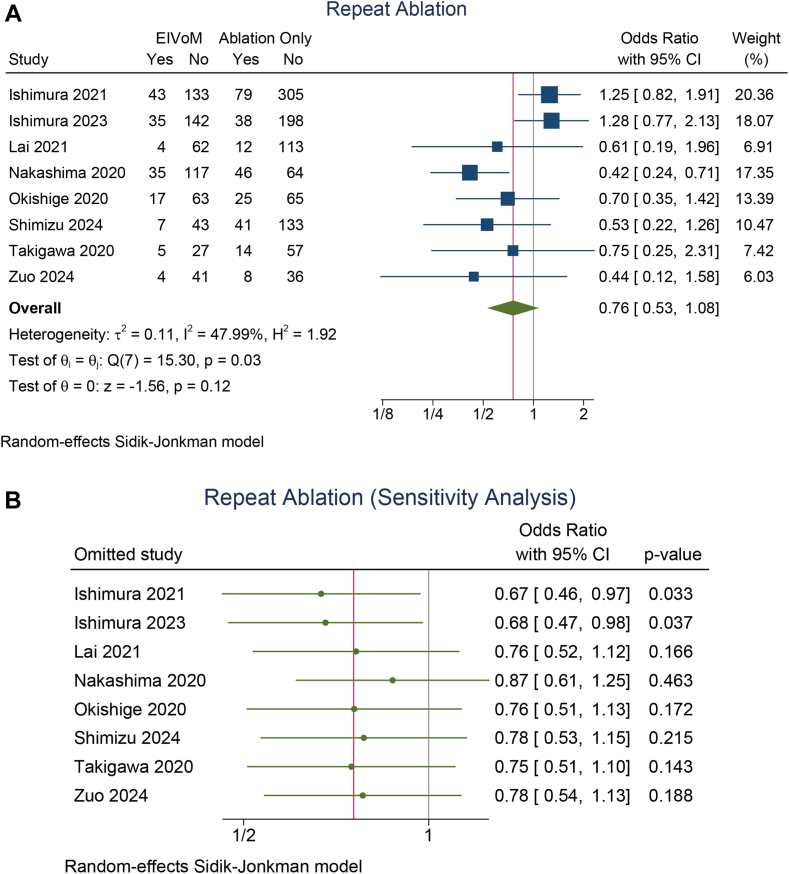

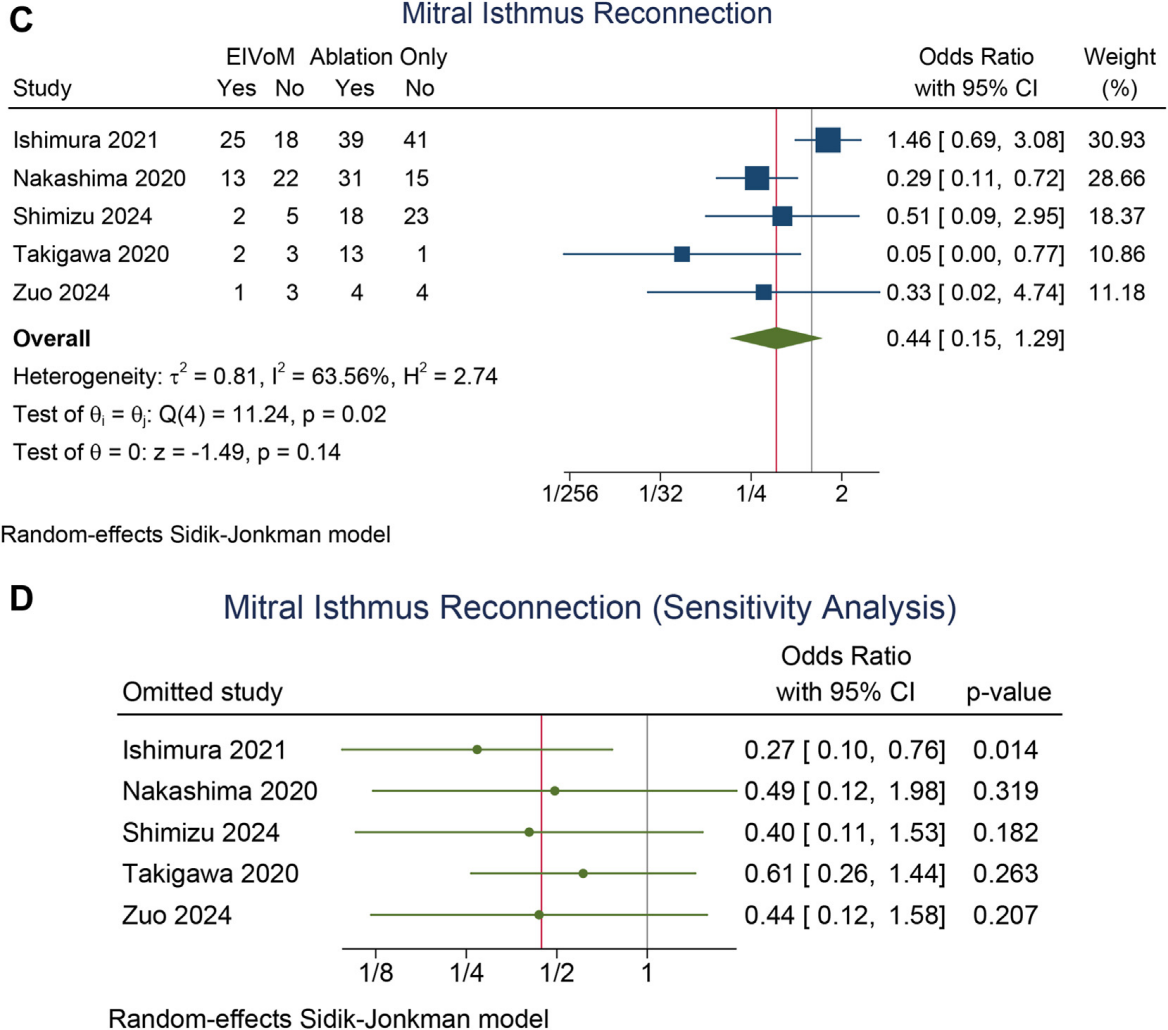
Repeat ablation and mitral isthmus reconnection on follow-up. (**A**) Repeat ablation between the EIVoM and ablation-only groups, (**B**) sensitivity analysis on repeat ablation, (**C**) mitral isthmus reconnection between the EIVoM and ablation-only groups, and (**D**) sensitivity analysis on mitral isthmus reconnection. CI = confidence interval; EIVoM = ethanol infusion into the vein of Marshall.

The rate of repeat ablation on follow-up was similar in the EIVoM and ablation-only groups (OR 0.76; 95% CI 0.53–1.08; *P* = .12; I^2^ = 48, *P* = .03) (Figure 4A). A leave-one-out sensitivity analysis showed that the repeat ablation rate was lower in the EIVoM group upon removal of either the Ishimura 2021^25^ study (OR 0.67; 95% CI 0.46–0.97; *P* = .033) or the Ishimura 2023^16^ study (OR 0.68; 95% CI 0.47–0.98; *P* = .037) (Figure 4B). A meta-regression analysis showed that age (OR 1.14; 95% CI 1.01–1.28; *P* = .029; for each 1-year increase) and LA diameter (OR 1.09; 95% CI 1.00–1.19; *P* = .042; for each 1-mm increase) were inversely associated with the benefits of EIVoM in terms of repeat ablation. A meta-regression analysis showed that LVEF did not significantly influence the rate of repeat ablation (*P* = .326).

The rate of mitral isthmus reconnection (OR 0.44; 95% CI 0.15–1.29; *P* = .14; I^2^ = 63.6, *P* = .02) (Figure 4C) was also similar in the EIVoM and ablation-only groups. A leave-one-out sensitivity analysis showed that the mitral isthmus reconnection rate was lower in the EIVoM group upon removal of the Ishimura 2021 study (OR 0.27; 95% CI 0.10–0.77; *P* = .014) (Figure 4D). A meta-regression analysis showed that age (OR 1.67; 95% CI 1.20–2.33; *P* = .003; for each 1-year increase) were inversely associated with the benefits of EIVoM in terms of mitral isthmus reconnection rate. A meta-regression analysis showed that LVEF did not significantly influence the rate of mitral isthmus reconnection (*P* = .261). LA diameter was reported by only 3 studies; thus, meta-regression analysis was not performed.

### Publication bias

**Figure 5:**
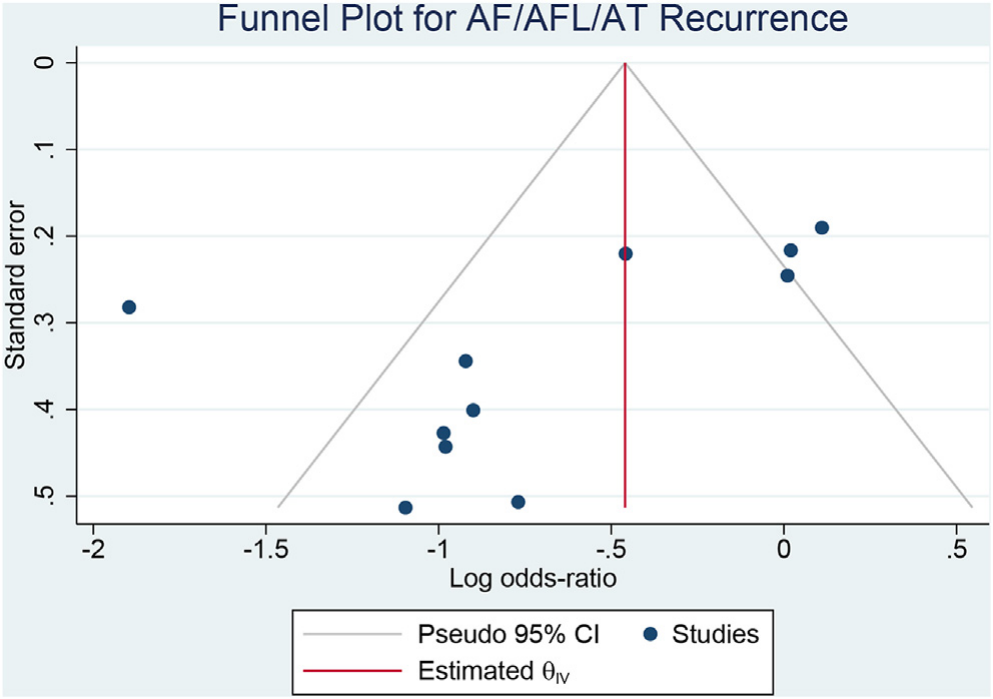
Funnel plot analysis for atrial tachyarrhythmia recurrence. θ_IV_ = inverse variance; AF = atrial fibrillation; AFL = atrial flutter; AT = atrial tachycardia; CI = confidence interval.

The regression-based Egger test showed no significant small study effects for ATa recurrence outcome (*P* = .058). Funnel plot analysis showed an asymmetrical distribution with an outlier favoring EIVoM, indicating possible publication bias (Figure 5).

### Subgroup analysis

A subgroup analysis for prospective observational studies showed that EIVoM reduced ATa recurrence compared with RF ablation-only (OR 0.52; 95% CI 0.30–0.90; I^2^ = 57.1%). The rate of MIBB in the EIVoM group was significantly higher than that in the ablation-only group (OR 4.27; 95% CI 1.89–9.71; I^2^ = 7.5%).

A subgroup analysis for randomized controlled trials showed that EIVoM reduced ATa recurrence compared with RF ablation-only (OR 0.60; 95% CI 0.39–0.91; I^2^ = 4.2%). The rate of MIBB in the EIVoM group was similar to that in the RF ablation-only group (OR 2.03; 95% CI 0.13–33.04; I^2^ = 96%).

## Discussion

This meta-analysis showed that EIVoM in addition to catheter ablation increased MIBB during the procedure and reduced ATa recurrence. Current evidence attained from this meta-analysis suggests that EIVoM did not reduce the number of repeat ablation procedures or mitral isthmus reconnections on follow-up; however, it was not statistically robust and may change. In addition, there was significant heterogeneity in the pooled effect estimates. Age and LA diameter were inversely associated with EIVoM benefits in terms of repeat ablation and mitral isthmus reconnection.

VoM has been shown to harbor the source of AF triggers and the tract for parasympathetic and sympathetic innervation that influence atrial tissue and contribute to AF maintenance.^5^, ^6^, ^7^, ^8^, ^9^ VoM has an epicardial course that may not be effectively targeted by endocardial ablation.^26^ Ethanol infusion may cause the elimination of AF triggers, atrial denervation, block at the mitral isthmus, and possibly other areas beyond the pulmonary vein.^5^^,^^12^, ^13^, ^14^, ^15^ This meta-analysis has shown that ethanol infusion reduced the number of ATa recurrences compared with ablation alone.

This meta-analysis showed that EIVoM did not reduce the number of repeat ablation procedures or mitral isthmus reconnections on follow-up. However, in sensitivity analysis, the removal of the Ishimura 2021 or Ishimura 2023 study resulted in significantly reduced mitral isthmus reconnection and repeat ablation in the EIVoM group. This indicates that the statistical results for these outcomes were not robust and may change with additional studies, which may support EIVoM use. The Ishimura 2023 study showed that ATa recurrence in EIVoM with an ethanol injection of ≥5 mL was reduced compared with an ethanol injection of <5 mL (25% vs 42%; *P* < .05), and ethanol injection was ≥5 mL in 60% of the patients. The Ishimura 2023 study also showed that patients receiving an ethanol injection of ≥5 mL require less debulking ablation to complete LA posterior wall isolation compared with those receiving an ethanol injection of <5 mL. Meta-regression analysis indicates that the benefits of EIVoM in terms of repeat ablation decreased as LA diameter increased. Patients in the Ishimura 2021 and Ishimura 2023 studies were older and had the largest LA diameter compared to those in other studies. The Ishimura 2021 study did not find a predictor of mitral isthmus reconnection on follow-up; however, we observed that the trend for mitral isthmus reconnection was similar to that for repeat ablation. Thus, it is likely that age and LA diameter influenced the possibility of mitral isthmus reconnection. In addition, the willingness of the patient or physician to pursue repeat ablation may have affected the results. Another limitation is the potential bias in the data on repeat ablation and mitral isthmus reconnection, as this would have been evaluated only in patients who underwent repeat ablation, which is a clinical decision.

Most studies included in this meta-analysis used EIVoM first followed by RF ablation, rather than RF ablation followed by adjunctive EIVoM. Gillis et al^27^ showed that EIVoM first can reduce the number of required RF applications, although the final incidence of block remained similar. Du et al^28^ demonstrated that performing EIVoM before RF ablation reduced the mitral isthmus ablation time and led to less ATa recurrence in a multivariate analysis (hazard ratio 0.25; 95% CI 0.07–0.92; *P* = .037). In the study by Gao et al,^24^ both EIVoM first (ATa recurrence 25%) and ablation first (ATa recurrence 21.4%) had lower ATa recurrence than did ablation-only (ATa recurrence 43.8%). Gao et al recommended performing EIVoM first because acute tissue edema and VoM stenosis due to ablation may render VoM not visualizable, which happened in their cohort. In addition, acute tissue edema may impede repeat ablation after a failed first pass; by performing EIVoM first, the tissue edema can be minimized and RF energy can be effectively delivered at sites of residual conduction. The argument to perform ablation first concerns the durability of EIVoM at the lesion border; studies have shown that endocardial and epicardial gaps were not covered or at the lesion border.^21^ Thus, EIVoM can mask important substrates to be ablated because of the low-voltage area induced by EIVoM.^21^^,^^25^, ^26^, ^27^^,^^29^ By performing ablation first guided by local potentials, it was hypothesized to reduce future reconnections in important areas. Considering the available comparative evidence on EIVoM or ablation first, the available data support the use of EIVoM followed by RF ablation. Since we observed that LA diameter potentially reduced the benefits in terms of repeat ablation, perhaps we can consider extensive mapping and then performing ablation first before proceeding with EIVoM in patients with large LA diameter to identify important pathways to be ablated and avoid its masking. However, more studies are needed for definitive conclusions.

### Clinical implications

Considering the findings of this meta-analysis and weighing on the limitations, EIVoM should be considered to obtain MIBB and reduce ATa recurrence, especially in nonparoxysmal AF, in which AF recurrence after PVI remained high. Whether EIVoM or ablation should be performed first was controversial; however, on the basis of limited available data, we can consider performing EIVoM first apart from patients with large LA diameter or advanced age or in a limited resource setting, although more research is needed to address this issue. The data on ethanol dose were inadequate to confidently draw a conclusion; however, an ethanol injection of ≥5 mL might be required, especially if LA posterior wall isolation is to be performed.

### Limitations

There were a limited number of randomized controlled trials, and most of the studies were observational. There are studies that may have overlapping samples, such as Ishimura 2021 and Ishimura 2023. However, the significance of this overlap is unclear because the Ishimura 2021 study did not specify the percentage of patients who underwent left atrial posterior wall isolation, while the Ishimura 2023 study indicated that all patients also underwent left atrial posterior wall isolation. In addition, the Ishimura 2023 study included an additional 3 years of EIVoM enrollment. Because of the limited number of studies and the availability of certain variables, meta-regression analysis cannot be performed to explore possible factors. Not all studies reported the mean dose of ethanol used and analysis categorized on the basis of ethanol dose; thus, dose-response analysis cannot be performed.

## Conclusion

This meta-analysis showed that EIVoM in addition to catheter ablation increased MIBB during the procedure and reduced ATa recurrence. Available evidence suggested that EIVoM did not reduce the number of repeat ablation procedures or mitral isthmus reconnections on follow-up. Age and LA diameter were inversely associated with EIVoM benefits in terms of repeat ablation and mitral isthmus reconnection.

## Disclosures

The authors have no conflicts to disclose.
